# Optimizing diagnostic methods and stem cell transplantation outcomes in pediatric bone marrow failure: a 50-year single center experience

**DOI:** 10.1007/s00431-023-05093-y

**Published:** 2023-07-13

**Authors:** Lotte Vissers, Mirjam van der Burg, Arjan Lankester, Frans Smiers, Alexander Mohseny

**Affiliations:** Department of Pediatric Hematology and Stem Cell Transplantation Unit, Willem-Alexander Children’s Hospital, Leiden University Medical Center, Leiden, The Netherlands

**Keywords:** Cytopenia, Aplastic anemia, Bone marrow failure, Hematopoietic stem cell transplantation, Immune suppressive therapy, Molecular analysis

## Abstract

Peripheral blood cytopenia, a frequent presenting symptom in pediatric patients, can be caused by bone marrow failure (BMF). Timely identification of patients with non-reversible BMF is of crucial importance to reduce the risks of invasive infections and bleeding complications. Most pediatric patients with severe persistent cytopenia, independent of the underlying cause, are offered allogeneic hematopoietic stem cell transplantation (HSCT) as curative therapy. Here we report on our management guidelines and HSCT outcomes of pediatric BMF patients to pinpoint improvements and future challenges. We formulated recommendations based on this 50 years’ experience, which were implemented at our center in 2017. By analysis of the HSCT cohort of 2017–2023, the 5-year outcome data is presented and compared to historical outcome data. In addition, outcomes of patients transplanted for identified inherited bone marrow failure syndromes (IBMFS) are compared to severe aplastic anemia (SAA) outcomes to underline the often multiorgan disease in IBMFS with implications for long-term survival. Survival of pediatric patients with irreversible BMF has improved tremendously. SAA patients transplanted after 2017 had a superior 5-year overall (OS) and event-free survival (EFS) of 97% and 85% compared to 68% and 59% in the cohort transplanted before 2017 (p = 0.0011 and p = 0.017). A similar trend was seen for BMF, with an OS and EFS of 89% for those transplanted after 2017 compared to 62% and 59% (p > 0.05). This improvement is mainly related to better survival in the first months after HSCT. The long-term survival after HSCT is lower in IBMFS patients as compared to SAA patients due to secondary malignancies and multiorgan toxicity.

*Conclusion*: Unbiased protocolized in-depth diagnostic strategies are crucial to increase the frequency of identifiable causes within the heterogeneous group of pediatric BMF. A comprehensive approach to identify the cause of BMF can prevent treatment delay and be useful to tailor treatment and follow-up protocols.

**Table:** 

**What is Known:** *• Irreversible BMF in pediatric patients can be caused by a wide spectrum of underlying diseases including (pre)malignant disease, IBMFS and AA. Identifying the exact underlying cause of BMF is crucial for tailored therapy, however often challenging and time-consuming.* *• Frontline allogeneic HSCT is offered to most pediatric patients with severe BMF as curative treatment.*	
**What is New:** *• Protocolized unbiased diagnostics, short time to treatment (< 3 months) and maximal supportive care until curative treatment can prevent complications with a negative effect on survival such as infection and bleeding.* *• Personalized follow-up protocols for IBMFS patients are essential to prevent a second decline in survival due to long-term treatment toxicity and extra-hematological disease complications.*

**Supplementary Information:**

The online version contains supplementary material available at 10.1007/s00431-023-05093-y.

## Introduction

Non-reversible bone marrow failure (BMF) in pediatric patients is caused by a broad spectrum of underlying diseases, including inherited bone marrow failure syndromes (IBMFS), (pre)malignant disease, and (idiopathic) aplastic anemia (AA) [1–3]. In up to 50% of these patients, a germline genetic defect can be identified causing BMF [4–6]. The etiology of IBMFS differs depending on the genetic defect or affected pathway. Well-characterized IBMFS include Fanconi Anemia (FA), caused by impaired DNA repair, Diamond-Blackfan Anemia (DBA) and Shwachman-Diamond Syndrome (SDS), associated with ribosome biogenesis defects, Severe Congenital Neutropenia (SCN) which is the result of deficient maturation of neutrophils and Dyskeratosis Congenita (DC) associated with inadequate telomere maintenance, resulting in shortened telomeres. In addition to cytopenia, patients with IBMFS often show multiorgan extra-hematological defects and have an increased risk for cancer, especially secondary myelodysplastic syndrome (MDS) and acute myeloid leukemia (AML) [1, 7]. Also independent of IBMFS, as a result of clonal hematopoiesis, MDS can develop and cause BMF [8]. Refractory cytopenia in childhood (RCC) is the most common subtype of MDS in children, characterized by less than 2% blasts in the peripheral blood and bone marrow [8–10]. The remainder of patients with BMF with unknown etiology is most often diagnosed as idiopathic AA [4]. Although the exact mechanism of AA is unknown, it is generally accepted to be caused by immune dysregulation [11, 12].

**Fig. 1:**
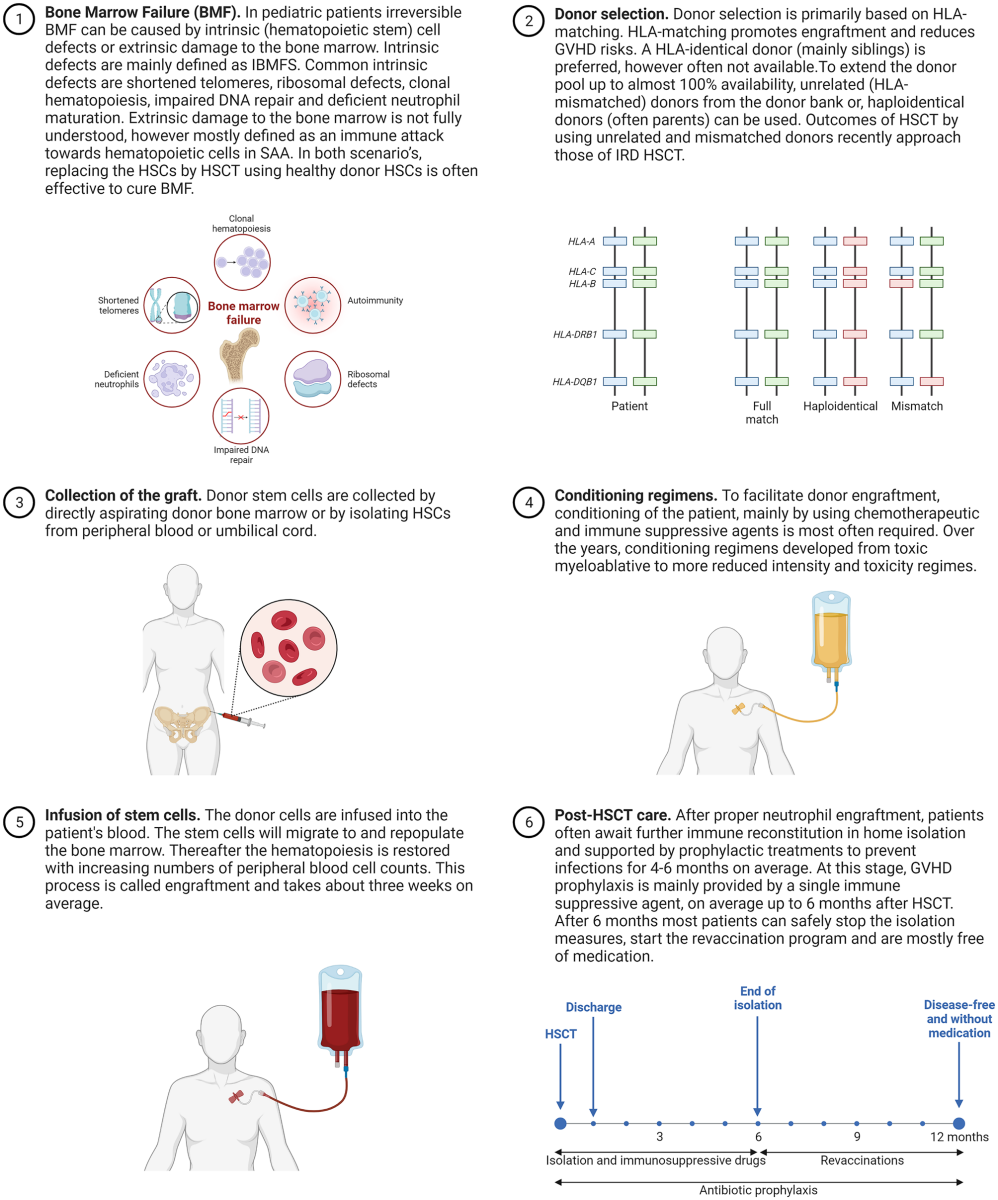
Visual dictionary of the key elements involved in pediatric bone marrow failure and allogeneic hematopoietic stem cell transplantation. BMF; bone marrow failure, IBMFS; inherited bone marrow failure syndromes, HSCs; hematopoietic stem cells, SAA; severe aplastic anemia, IRD; identical related donor, GVHD; graft versus host disease, HSCT; hematopoietic stem cell transplantation

Timely recognition of (irreversible) BMF and identification of the underlying cause of BMF result in reduced risks of invasive infections and bleeding complications. This requires guidelines for a consistent diagnostic approach for pediatric patients suspected of BMF. In these guidelines, in addition to morphological and histological examination of bone marrow aspirates and biopsies, molecular techniques and functional assays are of increasing value. In general, genetic screening is initiated in case of extra-hematological physical abnormalities and/or a positive family history [4, 13]. However, these aspects are not always identifiable at the onset of BMF, especially in young children. As a result, patients might be diagnosed as AA. Therefore, we proposed an extensive unbiased diagnostic algorithm [4]. This method includes broad genetic analysis and telomere length analysis in addition to the conventional diagnostic tests to achieve swift interpretation of first-line diagnostics and a higher yield of identifiable defects underlying irreversible BMF. As a result, the cause of BMF in pediatric patients was more often discovered. This allowed for risk-adapted organ and cancer monitoring, family counseling and prompt initiation of a curative treatment regime, mainly by allogeneic hematopoietic stem cell transplantation (HSCT, Fig. 1). In addition, the remaining group of BMF with unknown origin or etiology, classified as AA, became smaller. This provides the opportunity to stratify a group of AA for upfront immune suppressive therapy (IST) treatment with expected superior therapy efficacy and provides a better homogeneous group for future research to explore AA etiology and pathogenesis.

Pediatric BMF patients are transplanted in our center for over 50 years. The first severe AA (SAA) patient (BMF of unknown origin at the time) was transplanted in 1971 and the first patient with severe BMF due to FA was transplanted in 1972. During the years following, conditioning regimens developed from toxic myeloablative to more reduced intensity and toxicity regimes (Supplementary Table 1). Especially for the IBMFS group, chemotherapy regimens aimed at less organ toxicity. While patients transplanted by using bone marrow (BM) from HLA-identical sibling donors showed superior outcomes, delay in HSCT or second-line HSCT after upfront IST, both resulted in higher mortality in pediatric patients [14]. The introduction of post-transplantation cyclophosphamide (PT-Cy) to prevent graft versus host disease (GVHD) provided access to a great number of (mismatched) unrelated donors. In the following years, outcomes of HSCT by using (mismatched) unrelated donors, increasingly improved and are currently comparable with the success rates of HLA-matched transplantation.

## Methods

### Design

**Fig. 2:**
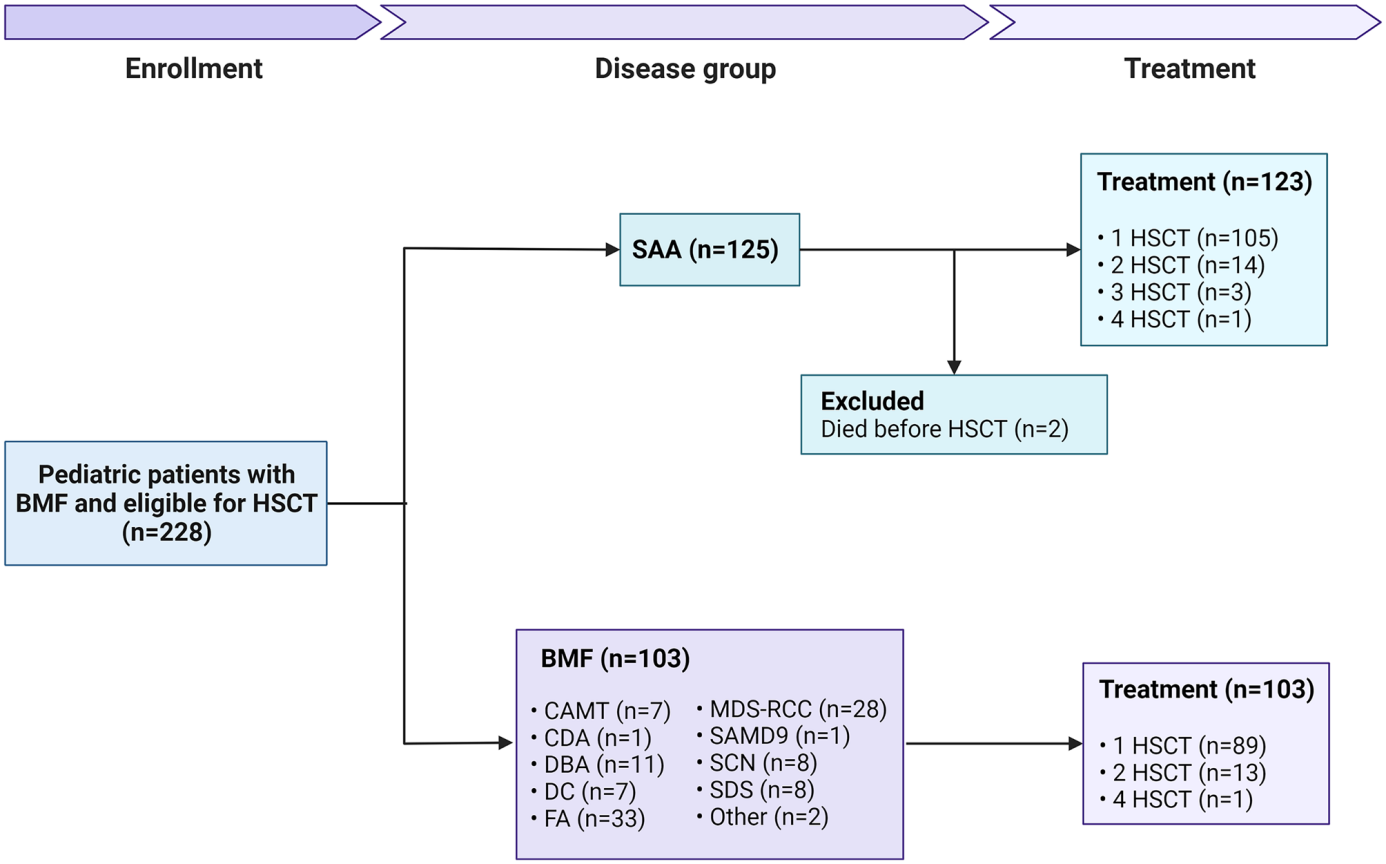
Study flow diagram. In total, 228 pediatric patients with bone marrow failure were included. HSCT; hematological stem cell transplantation, BMF; bone marrow failure, SAA; severe aplastic anemia, CAMT; Congenital amegakaryocytic thrombocytopenia, CDA; congenital dyserythopoietic anemia, DBA; Diamond-Blackfan anemia, DC; Dyskeratosis congenita, FA; Fanconi anemia, MDS-RCC; Myelodysplastic syndrome-refractory cytopenia of childhood, SCN; sever congenital neutropenia, SDS; Shwachman-Diamond syndrome

For this retrospective cohort study, data was obtained from the Bone Marrow Transplantation Database at the Leiden University Medical Center (LUMC). This database includes the clinical data of all pediatric patients who received an HSCT in the LUMC as of 1965. MDS or malignant disorders causing BMF, other than MDS-RCC were not included in the analysis. Between 1971 and 2022 a total of 265 transplantations were performed in 226 pediatric patients with BMF (Fig. 2). For the purpose of this study, this group was divided in 1. HSCT for BMF due to an identified cause or underlying disease (categorized as BMF group, n = 103) and 2. HSCT for BMF of unknown origin (categorized as SAA group, n = 125). For patients who were treated with more than one HSCT, follow-up data from the first HSCT was used. 5-years follow-up outcome data of patients transplanted from 2017 was compared to the historical cohort between 1971–2017.

### Data collection

The following data were collected: diagnosis, patient characteristics, donor characteristics, transplantation characteristics, complications and outcomes such as: overall survival (OS), event-free survival (EFS) and GVHD. EFS or OS was defined as date from initial diagnose to date of relapse, second HSCT and/or death by any cause. Patient characteristics include age and gender. Donor characteristics cover donor type (HLA-matched related, HLA-mismatch related and unrelated donor (UD) including both matched and mismatched donors) and source (BM, peripheral blood stem cells (PBSC) or umbilical cord blood (UCB). Transplantation characteristics include conditioning regimen, GVHD prophylaxis and the number and date of transplantation. Missing data were collected from the electronical patient records by the treating physician.

### Statistical analysis

Statistical analysis was performed by using RStudio (R4.2.2). Characteristics of the patients and donors were described. For analyzing continuous variables among the cohorts, the Wilcoxon rank sum test was used. Pearson’s Chi-squared test was used for categorical data. For the survival analyses, Kaplan-Meier curves were plotted including 95% confidence intervals. Upon last date of follow-up, patients without an event were censored. The 5-year OS and EFS were compared between the cohorts transplanted before and after 2017 using a Log-rank test. In addition, long-term (30-year) OS and EFS of patients transplanted before 2017 was visualized. 5-year OS per donor type was compared using Kaplan-Meier curves and a Long-rank test. Median follow-up time was computed using the reversed Kaplan-Meier method. Endpoint of this study is 01-01-2023.

## Results

### Patient characteristics

The clinical characteristics of the patients included are summarized in Table 1 and Fig. 2. In the BMF group (n = 103), 89 patients received one transplantation, 13 patients needed a second transplantation and 1 patient received four transplantations (total of 119 transplantations). In the SAA group, 2 patients died before HSCT and were therefore excluded from analysis. Of the remaining (n = 123), 105 patients received one transplantation, 14 patients needed a second transplantation, 3 patients were transplanted for a third time and 1 patient received four transplantations (total of 146 transplantations, Fig. 2). A significantly younger age at HSCT was observed for patients in the BMF group transplanted after 2017 (p = 0.016). In contrast, for the SAA group, the average age at HSCT did not differ between the cohort after 2017 and the historical cohort. Patients transplanted before 2017 had a significantly longer follow-up. In comparison to the historical cohort, after 2017 a significantly greater proportion of the patients was transplanted by using an UD (p = 0.002). Sex and stem cell source did not significantly differ between the cohorts. BM was used for the majority (90%) of the transplantations.

**Table 1:** Patient characteristics

**Characteristics**	SAA			BMF		
	** < 2017**, N = 80	** ≥ 2017**, N = 43	**p-value**	** < 2017**, N = 86	** ≥ 2017**, N = 17	**p-value**
Age at HSCT (y), median (IQR)	10.5 (6.4, 13.6)	10.2 (5.3, 13.7)	> 0.9	7.3 (4.6, 10.9)	5.3 (2.5, 6.5)	0.016
Sex, n (%)			0.8			0.4
Female	35 (44%)	18 (42%)		32 (37%)	8 (47%)	
Male	45 (56%)	25 (58%)		54 (63%)	9 (53%)	
Follow-up time (m), median (min–max)	314 (74–591)	38 (2–72)	< 0.001	219 (73–447)	35 (7–67)	< 0.001
Diagnosis, n (%)						
SAA	80 (100%)	43 (100%)				
BMF				86 (100%)	17 (100%)	
CAMT				4 (4.7%)	3 (18%)	
CDA				1 (1.2%)	0 (0%)	
DBA				9 (10%)	2 (12%)	
DC				6 (7.0%)	1 (5.9%)	
FA				33 (38%)	0 (0%)	
MDS-RCC				24 (28%)	4 (24%)	
SAMD9 mutation				0 (0%)	1 (5.9%)	
SCN				4 (4.7%)	4 (24%)	
SDS				3 (3.5%)	2 (12%)	
Other				2 (2.3%)	0 (0%)	
Donor relation, n (%)			0.002			> 0.9
Matched related	53 (66%)	16 (37%)		28 (33%)	6 (35%)	
Mismatch related	9 (11%)	4 (9.3%)		14 (16%)	2 (12%)	
Unrelated	18 (22%)	23 (53%)		44 (51%)	9 (53%)	
Stem cell source, n (%)			0.4			0.3
Bone marrow	75 (94%)	41 (95%)		71 (83%)	17 (100%)	
Cord blood	0 (0%)	1 (2.3%)		7 (8.1%)	0 (0%)	
Peripheral blood	5 (6.2%)	1 (2.3%)		8 (9.3%)	0 (0%)	

*SAA* severe aplastic anemia, *BMF* bone marrow failure, *CAMT* Congenital amegakaryocytic thrombocytopenia, *CDA* congenital dyserythopoietic anemia, *DBA* Diamond-Blackfan anemia, *DC* Dyskeratosis congenita, *FA* Fanconi anemia, *MDS-RCC* Myelodysplastic syndrome-refractory cytopenia of childhood, *SCN* sever congenital neutropenia, *SDS* Shwachman-Diamond syndrome

### Survival

**Fig. 3:**
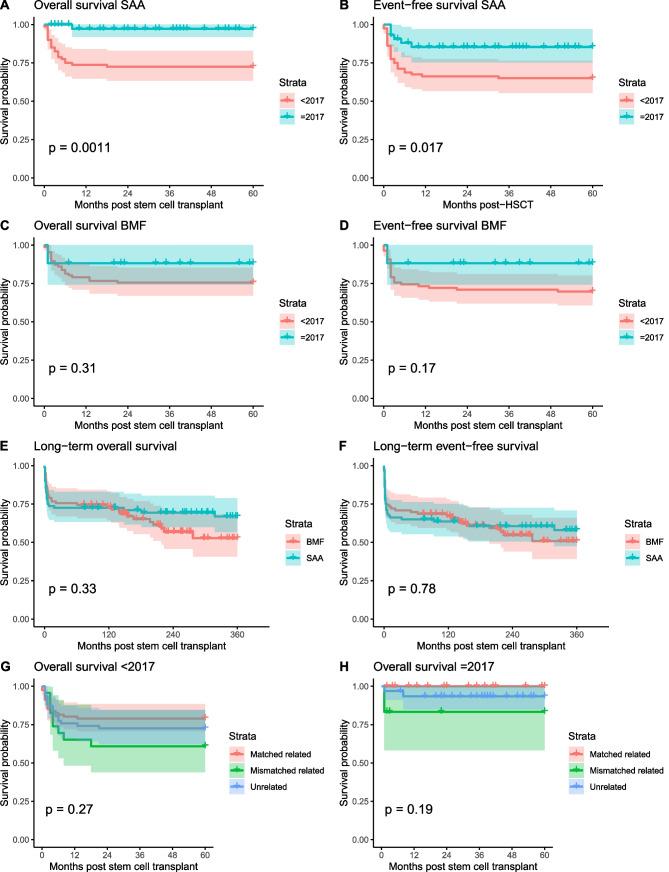
Survival outcomes. **A**, **B** SAA patients transplanted after 2017 have a significant higher 5-year OS and EFS. **C**, **D** Although not significant, a similar trend was observed indicating an improved survival for BMF patients transplanted after 2017. **E**, **F** 10 to 20 years post HSCT, a second decline in survival can be observed for BMF patients, which is not apparent for SAA patients. **G**, **H** For all donor types, treatment has been improved resulting in better survival outcomes after 2017. BMF; bone marrow failure, SAA; severe aplastic anemia

SAA patients transplanted after 2017 had a superior 5-year OS and EFS, respectively 97% and 85% as compared to 68% and 59% in the cohort transplanted before 2017 (p = 0.0011 and p = 0.017, respectively, Fig. 3A, B). The strongest decline in survival was observed within the first 3 months after HSCT. Although in the BMF group a similar trend was observed indicating improved survival in the cohort transplanted after 2017, the improvements in both OS and EFS were not statistically significant (p = 0.31 and p = 0.17, respectively, Fig. 3C, D). Moreover, by analyzing long-term follow-up data of patients transplanted before 2017 (Fig. 3E, F), in contrast to the SAA cohort, a second decline in survival of BMF patients 10 to 20 years after HSCT becomes apparent. Of the eleven late mortalities, cause of death was due to secondary malignancies in four patients (Supplementary Table 2). Six patients died due to multiorgan toxicity in combination with renal or pulmonal failure. For one patient, cause of death was unknown. Lastly, for all donor types, OS was better for patients transplanted after 2017 (Fig. 3H, G).

### Graft versus host disease

For both SAA and BMF groups, independent of the year of HSCT, acute (grade II-IV) and chronic GVHD incidences were low (acute GVHD grade II-IV 12.5% in SAA group vs 12.9% in BMF group, chronic limited GVHD 6.7% in SAA group vs 4.3% in BMF group and extensive chronic GVHD 5.7% in SAA group vs 6.4% in BMF group, Table 2). Patients transplanted in the BMF group after 2017 suffered less relevant GVHD (5.9% acute GVHD > grade I and 0% chronic GVHD, Table 2), however this observation was not significant mainly due to the small group size (n = 18).

**Table 2:** Graft versus host disease

	SAA		BMF	
	** < 2017**, N = 80	** ≥ 2017**, N = 43	** < 2017**, N = 86	** ≥ 2017**, N = 17
Acute GVHD				
No aGVHD	61 (79%)	28 (74%)	68 (81%)	11 (65%)
Grade I	7 (9.1%)	5 (13%)	4 (4.8%)	5 (29%)
Grade II-IV	9 (12%)	5 (13%)	12 (14%)	1 (5.9%)
Unknown	3	5	2	0
Chronic GVHD				
No cGVHD	56 (84%)	36 (95%)	68 (87%)	16 (100%)
Limited	6 (9.0%)	1 (2.6%)	4 (5.1%)	0 (0%)
Extensive	5 (7.5%)	1 (2.6%)	6 (7.7%)	0 (0%)
Unknown	13	5	8	1

*SAA* severe aplastic anemia, *BMF* bone marrow failure, *GVHD* graft versus host disease, *aGHVD* acute graft versus host disease, *cGHVD* chronic graft versus host disease

## Discussion

Managing pediatric patients with BMF is challenging at all stages of the care pathway. An individualized treatment and surveillance plan will depend on the underlying cause of the BMF and the severity of the condition. Identification of the causative defects underlying BMF is particularly difficult in otherwise healthy children without extra-hematological signs and symptoms. The caveat there is that these patients are categorized as AA. Given the lack of specific disease markers for AA, the diagnosis remains a diagnosis per exclusion. Recent advances in genomic evaluation including whole exome next-generation sequencing and functional analysis such a telomere length analysis, provide the opportunity for an unbiased diagnostic approach [4]. Concordant genetic and functional analyses provide the opportunity to detect novel genetic variations and to define genetic variations of unknown significance (VUS). Identification of the cause of BMF delivers crucial information for treatment strategies and monitoring protocols on individual patient level. Increased diagnostic yield of identified causes of BMF also results in a smaller remnant group to be diagnosed as AA. The increased homogeneity of this group can result in better outcomes of IST and provides a better-defined group for future AA related research. In addition, the unbiased diagnostic approach provides the basic information for crucial insights in understanding DNA repair, telomere and ribosome biology, and hematopoietic stem cell and stromal niche regulation.

Risk assessment of malignant transformation represents another challenge at the diagnostic stage. Based on morphological and histological characteristic changes defined as dysplasia, efforts are directed towards the differentiation between hypoplastic bone marrow at risk for clonal evolution and malignant transformation (such as in MDS-RCC) and hypoplastic bone marrow without increased risk of malignant transformation (such as in SAA) [15–17]. Also here, molecular diagnostics can be of crucial importance. Next to the evaluation of clonal hematopoiesis driven by well-known MDS/AML related cytogenetic alterations, somatic mutations might be predictive of malignant transformation [18, 19]. In addition, increasing evidence is available that clonal hematopoiesis does not equate malignant transformation on itself [20]. In particular situations, such as with monosomy 7 in *SAMD-9* or *GATA-2 germline* mutation driven BMF, clonal hematopoiesis may even be part of an escape mechanism to restore hematopoiesis and should not require immediate definitive treatment to prevent malignant transformation. All in all, molecular bone marrow analysis beyond the frequent cytogenetic changes and expert opinion are required to identify markers of clonal evolution and malignant transformation for an individualized monitoring plan of BMF patients at risk.

Also, the treatment of pediatric BMF patients is accompanied with challenges. The majority of these patients present with or develop severe cytopenia or risk indicators for hematologic malignancies. Thereby for most patients, HSCT is an attractive and often the only curative treatment option. Based on our long-term experience in HSCT for pediatric patients with BMF as presented here, we draw the following conclusions. HSCT has an unequaled curative potential to restore BM defects independent of the underlying cause. Moreover, with the current successes of HLA-mismatched transplantations by effective GVHD prevention such as PT-Cy or alpha/beta T-cell depletion, and reduced toxicity conditioning regimens, HSCT is increasingly offered as the first choice of treatment to pediatric BMF patients. However, short- and long-term (event-free) survival rates are evidently dependent on the management of the patients before and after transplantation. A structured multidisciplinary approach directed towards unbiased but swift diagnostics at the suspicion to diagnosis stage together with maximal supportive care to prevent infections and bleeding result into significant gain of survival rates within the first 3 months after the onset of the disease. This is underlined by the significant improved overall and disease-free survival rates of BMF patients transplanted after 2017 independent of the underlying cause. In addition, especially in patients with IBMFS, protocolized approaches to systematically screen for late effects post HSCT are of crucial importance. The second decline in survival of these patients 10 to 20 years after HSCT, often explained by secondary malignancies, lung- and liver fibrosis and other organ failures, underlines the essence of personalized follow-up protocols. This wide variety of long-term toxicities including chronic GVHD, delayed or unbalanced immune reconstitution, iron overload, developmental delay and psychosocial impairment and malignant disease require a multidisciplinary approach guided by centers of expertise [21].

### Supplementary Information

Below is the link to the electronic supplementary material.

Supplementary file1 (DOCX 21 KB)

## List of abbreviations

AA: Aplastic anemia
AML: Acute myeloid leukemia
BM: Bone marrow
CAMT: Congenital Amegakaryocytic Thrombocytopenia
CDA: Congenital Dyserythropoietic Anemia
DBA: Diamond-Blackfan Anemia
DC: Dyskeratosis Congenita
FA: Fanconi Anemia
EFS: Event-free survival
GVHD: Graft versus host disease
HSCs: Hematopoietic Stem Cells
HSCT: Hematopoietic Stem Cell Transplantation
IBMFS: Inherited Bone Marrow Failure Syndromes
IST: Immune suppressive therapy
IRD: Identical Related Donor
LUMC: Leiden University Medical Center
MDS: Myelodysplastic syndrome
OS: Overall survival
PBSC: Peripheral blood stem cells
PT-Cy: Post-transplantation cyclophosphamide
RCC: Refractory cytopenia in childhood
SAA: Severe aplastic anemia
SCN: Severe Congenital Neutropenia
SDS: Shwachman-Diamond Syndrome
UD: Unrelated Donor
UCB: Umbilical cord blood
VUS: Variations of unknown significance
