# Supplementary material for: Optimizing diagnostic methods and stem cell transplantation outcomes in pediatric bone marrow failure: a 50-year single center experience
Source: Eur J Pediatr. 2023 Jul 13;182(9):4195–203. doi: 10.1007/s00431-023-05093-y (PMC10570154; doi:10.1007/s00431-023-05093-y)
Supplement: Supplementary file 1 — Supplementary file1 (DOCX 21 KB) [file 431_2023_5093_MOESM1_ESM.docx]

**Supplementary information**

***Supplementary table 1. Conditioning regimen over the years.*** *During the years, conditioning regimens developed from toxic myeloablative to more reduced intensity and toxicity regimes. A) Conditioning regimen for most SAA developed from more toxic to a rather immune-ablative conditioning regimen consisting of Flu/Cy without radiation. B) For IBMFS, individualized regimens were used depending on the exact diagnosis and acceptable toxicity.*

*Bu; Busulfan, Cy; Cyclophosphamide, Cyta; Cytarabine, Flu; Fludarabine, Mel; Melphalan, TAI; total abdominal irradiation, TBI; total body irradiation, Thio; Thiotepa, Treo; Treosulfan.*

***A)***

|  | **Bu/Cy** | **Bu/Cy/**  **Mel** | **Bu/Flu** | **Cy** | **Cy/Cyta/**  **TBI** | **Cy/Mel/**  **TAI** | **Cy/TAI** | **Cy/TBI** | **Cy/Thio/**  **TBI** | **Flu/Cy** | **Flu/Cy/**  **TAI** | **Treo/Flu** | **Treo/Flu/Thio** | **Unknown** |
| --- | --- | --- | --- | --- | --- | --- | --- | --- | --- | --- | --- | --- | --- | --- |
| **<1990** | 0 | 0 | 0 | 1 | 0 | 0 | 0 | 4 | 0 | 0 | 0 | 0 | 0 | 21 |
| **1990-1995** | 0 | 0 | 0 | 4 | 0 | 0 | 0 | 5 | 0 | 0 | 0 | 0 | 0 | 4 |
| **1995-2000** | 2 | 1 | 0 | 3 | 1 | 0 | 1 | 3 | 1 | 0 | 0 | 0 | 0 | 0 |
| **2000-2005** | 1 | 1 | 0 | 0 | 0 | 1 | 7 | 0 | 0 | 0 | 1 | 0 | 0 | 0 |
| **2005-2010** | 0 | 0 | 0 | 1 | 0 | 0 | 0 | 0 | 0 | 2 | 0 | 0 | 0 | 0 |
| **2010-2015** | 0 | 0 | 1 | 0 | 0 | 0 | 0 | 0 | 0 | 10 | 0 | 0 | 0 | 0 |
| **>2015** | 0 | 0 | 0 | 1 | 0 | 0 | 0 | 0 | 0 | 39 | 0 | 3 | 3 | 1 |

***B)***

|  | **Bu/Cy** | **Bu/Cy/**  **Flu** | **Bu/Cy/**  **Mel** | **Bu/Flu** | **Cy** | **Cy/Cyta/**  **TAI** | **Cy/Cyta/**  **TBI** | **Cy/TAI** | **Cy/TBI** | **Cy/Treo/**  **TAI** | **Flu/Cy** | **Flu/Cy/**  **TAI** | **Flu/Cy/**  **Thio** | **Thio/Flu** | **Treo/Flu** | **Treo/Flu/**  **Thio** | **Unknown** |
| --- | --- | --- | --- | --- | --- | --- | --- | --- | --- | --- | --- | --- | --- | --- | --- | --- | --- |
| **<1990** | 0 | 0 | 0 | 0 | 4 | 0 | 0 | 1 | 1 | 0 | 0 | 0 | 0 | 0 | 0 | 0 | 3 |
| **1990-1995** | 1 | 0 | 0 | 0 | 1 | 2 | 7 | 3 | 0 | 0 | 0 | 0 | 0 | 0 | 0 | 0 | 1 |
| **1995-2000** | 1 | 0 | 3 | 0 | 0 | 3 | 0 | 3 | 0 | 0 | 0 | 0 | 0 | 0 | 0 | 0 | 0 |
| **2000-2005** | 3 | 1 | 4 | 0 | 0 | 0 | 1 | 1 | 0 | 0 | 0 | 5 | 0 | 0 | 0 | 0 | 0 |
| **2005-2010** | 2 | 0 | 2 | 0 | 0 | 0 | 0 | 0 | 0 | 1 | 3 | 0 | 0 | 3 | 0 | 0 | 0 |
| **2010-2015** | 0 | 0 | 0 | 10 | 0 | 0 | 0 | 0 | 0 | 0 | 5 | 0 | 0 | 6 | 0 | 0 | 0 |
| **>2015** | 0 | 0 | 0 | 4 | 0 | 0 | 0 | 0 | 0 | 0 | 2 | 0 | 1 | 1 | 7 | 7 | 0 |

***Supplementary table 2. Cause of death of BMF with late mortality.*** *Within the BMF cohort, a second decline in survival can be seen around 10 to 20 years after HSCT. This decline is associated with secondary malignancies or multiorgan toxicity in combination with renal or pulmonal failure.*

*DC; Dyskeratosis congenita, CAMT; Congenital amegakaryocytic thrombocytopenia, FA; Fanconi anemia, HSCT; hematological stem cell transplantation,*

| **Diagnosis** | **Months between HSCT and death** | **Cause of death** |
| --- | --- | --- |
| DC | 117 | Multiorgan toxicity and pulmonal failure |
| DC | 124 | Multiorgan toxicity and renal failure |
| CAMT | 139 | Multiorgan toxicity and renal failure |
| DC | 140 | Secondary malignancy |
| FA | 154 | Secondary malignancy |
| FA | 166 | Multiorgan toxicity and renal failure |
| FA | 197 | Secondary malignancy |
| FA | 203 | Multiorgan toxicity and pulmonal failure |
| FA | 218 | Multiorgan toxicity and renal failure |
| FA | 223 | Secondary malignancy |
| FA | 277 | Unknown |
